# Supplementary material for: The role of integrins in Drosophila egg chamber morphogenesis
Source: Development. 2019 Dec 9;146(23):dev182774. doi: 10.1242/dev.182774 (PMC6918751; doi:10.1242/dev.182774)
Supplement: Supplementary information [file develop-146-182774-s1.pdf]

Supplemental Figures

Supp Figure 1

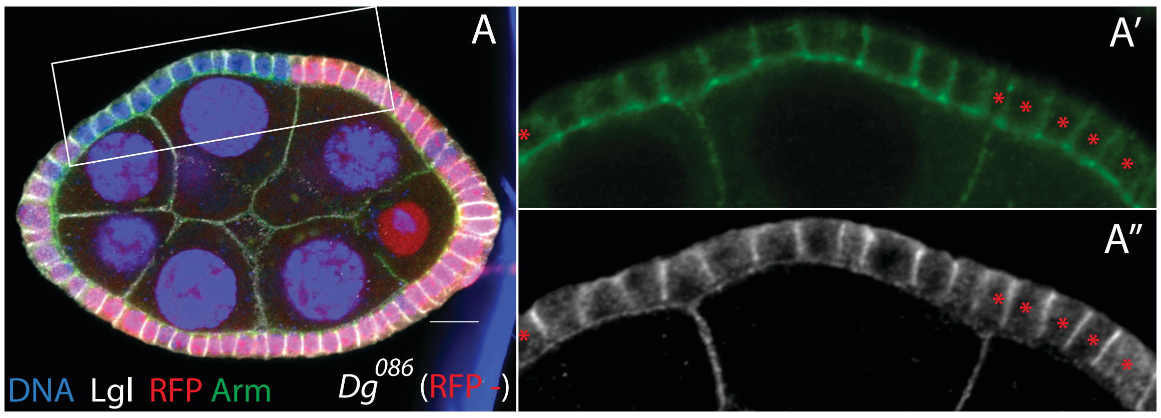

Figure S1

(A) A stage 7 egg chamber containing *Dg*^{086} mutant cells marked by loss of RFP (red) and stained for Armadillo (green) and Lgl (white). The mutant cells show neither epithelial disorganisation nor a disruption in apical-basal polarity when they occur along the sides of egg chambers (n=10)
Scale bar is 10μM.

Supplementary Figure 2

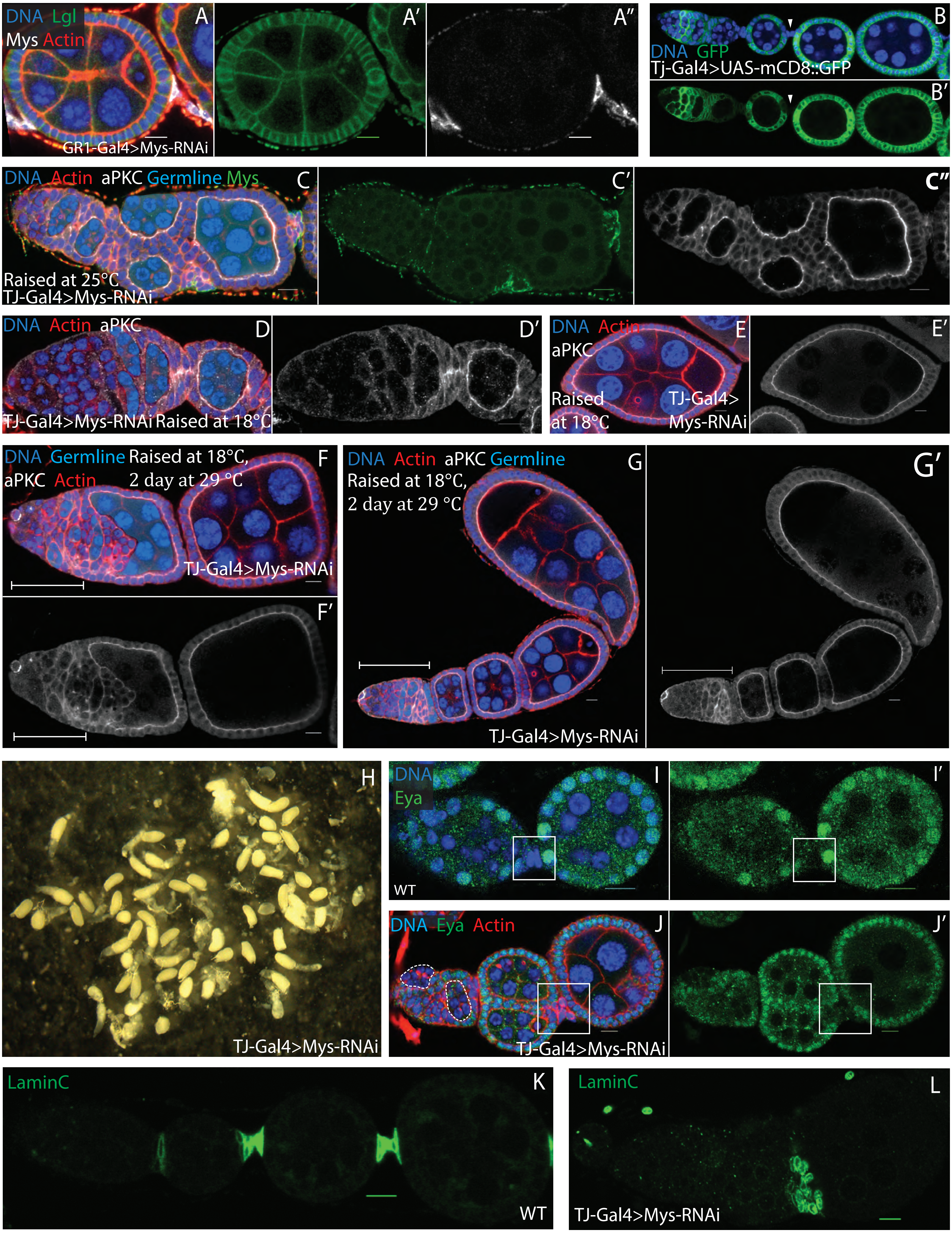

Development • Supplementary information

Figure S2

(A) A stage 4 egg chamber expressing UAS-*mys*-RNAi under the control of GR1-Gal4 stained for Mys (white), Lgl (green), F-actin (white) and DNA (blue). The flies were raised at 18°C and moved as adults to 29°C for 6 days before dissection. Although Mys is efficiently depleted from the follicle cells, this does not result in a loss of tissue organisation or apical-basal polarity (n=6).

(B) A germarium expressing mCD8-GFP (green) under the control of TJ-Gal4, which drives the expression of UAS constructs in all somatic cells of the ovary, apart from the interfollicular stalks (white arrow heads) (n=4).

(C) A germarium from flies raised at 25°C expressing UAS-*mys*-RNAi (Bloom 27735) under the control of TJ-Gal4, stained for Mys (green), aPKC (white), F-actin (red) and DNA (blue). Mys is efficiently knocked down in all somatic cells (except interfollicular stalk cells), resulting in gross disorganisation of the ovariole. The follicle cells that contact the germ line polarise correctly as shown by the apical localisation of aPKC (n=6/7).

(D and E) Germaria from TJ-Gal4; UAS-*mys*-RNAi (Bloom 27735) flies raised at 18°C. At this temperature, TJ-GAL4 does not drive sufficient *mys*-RNAi expression to produce a phenotype, resulting in normal germaria (D) (n=11) and egg chambers (E) (n=8)**.**

(F and G) Germaria from TJ-Gal4; UAS-*mys*-RNAi (Bloom 27735) flies raised at 18°C and then shifted to 29°C for two days before dissection, aPKC (white), F-actin (red) and DNA (blue). The early germine cysts are outlined in pale blue. Knock-down of Mys disrupts the organisation of the follicle cells in the germarium (brackets F and G) (n=20/20) but does not affect older egg chambers (n=19/20).

(H) Eggs from TJ-Gal4; UAS-*mys*-RNAi (Bloom 27735) flies raised at 25°C. Many of the eggs are misshapen and rounder than normal, consistent with a defect in egg chamber rotation (n=7).

(I) Eya staining in wild-type. Eya is expressed in the main body follicle cells, but not the polar or stalk cells (box) (n=1).

(J) An ovariole expressing UAS-*mys*-RNAi (Bloom 33647) under the control of TJ-Gal4 at 25°C, stained for Eya (green), F-actin (red) and DNA (blue). Eya negative cells can be found between developing germline cysts (box), indicating that stalk and polar cells have been specified normally. Early germline cysts are outlined with dashed lines (n=6).

All scale bars are 10μM.

Development • Supplementary information

Supp Figure 3

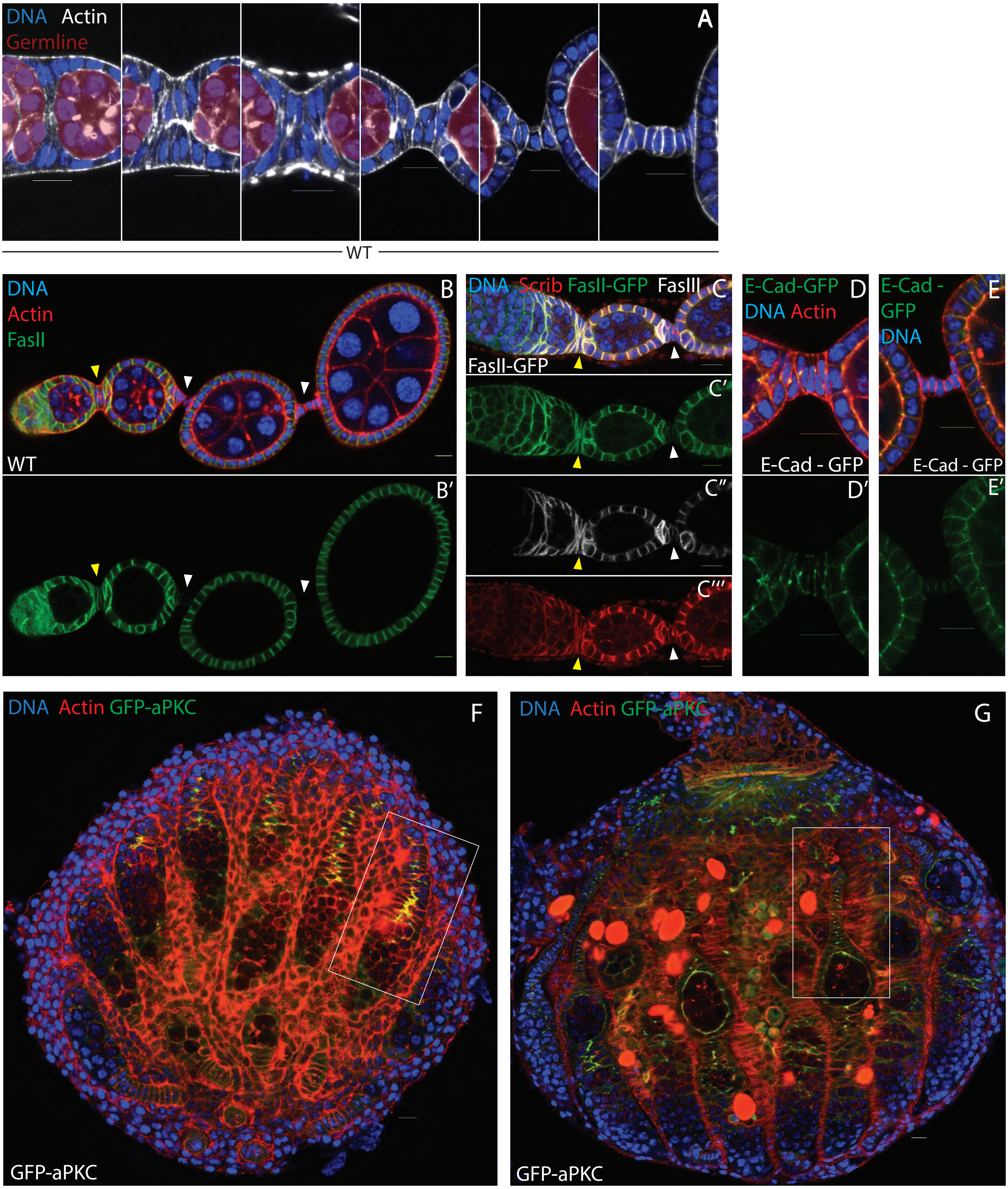

Development • Supplementary information

Figure S3

(A) Series of interfollicular stalks of increasing age (left to right) stained for F-actin (white) and DNA (blue). Interfollicular stalks are made from two rows of cells between adjacent germline cysts that intercalate to form a one cell wide stalk.

(B) A wild-type ovariole stained for FasII (green), F-actin (red) and DNA (blue). FasII localises laterally in follicle cells and the developing stalk (yellow arrowhead), but is down regulated later as the stalks mature (white arrowheads) (n=5).

(C) A wild-type ovariole expressing FasII-GFP (green) and stained for FasIII (white), Scribbled (red) and DNA (blue). FasIII is down-regulated as interfollicular stalks mature (yellow vs white arrow head), but at a later time point than FasII (compare white arrowheads in C' & C'') (n=3).

(D and E) Developing stalks expressing E-Cad-GFP (green) and stained for actin (red) and DNA (blue). Like aPKC, E-Cad localises to lateral punctae as the stalk cells intercalate (D) and is down-regulated as the stalk matures (E) (n=1).

(F) A developing pupal ovary expressing aPKC-GFP (green) and stained for actin (red) and DNA (blue). aPKC localises to the apical sides of the basal stalk cells prior to intercalation (n=2). Enlarged image of box is in Fig. 3I

(G) An older pupal ovary expressing aPKC-GFP (green) and stained for F-actin (red) and DNA (blue), aPKC relocalises to lateral punctae as the basal stalk cells intercalate (n=2). Enlarged image of box is in Fig. 3J.

All scale bars are 10μM.

Supp Figure 4

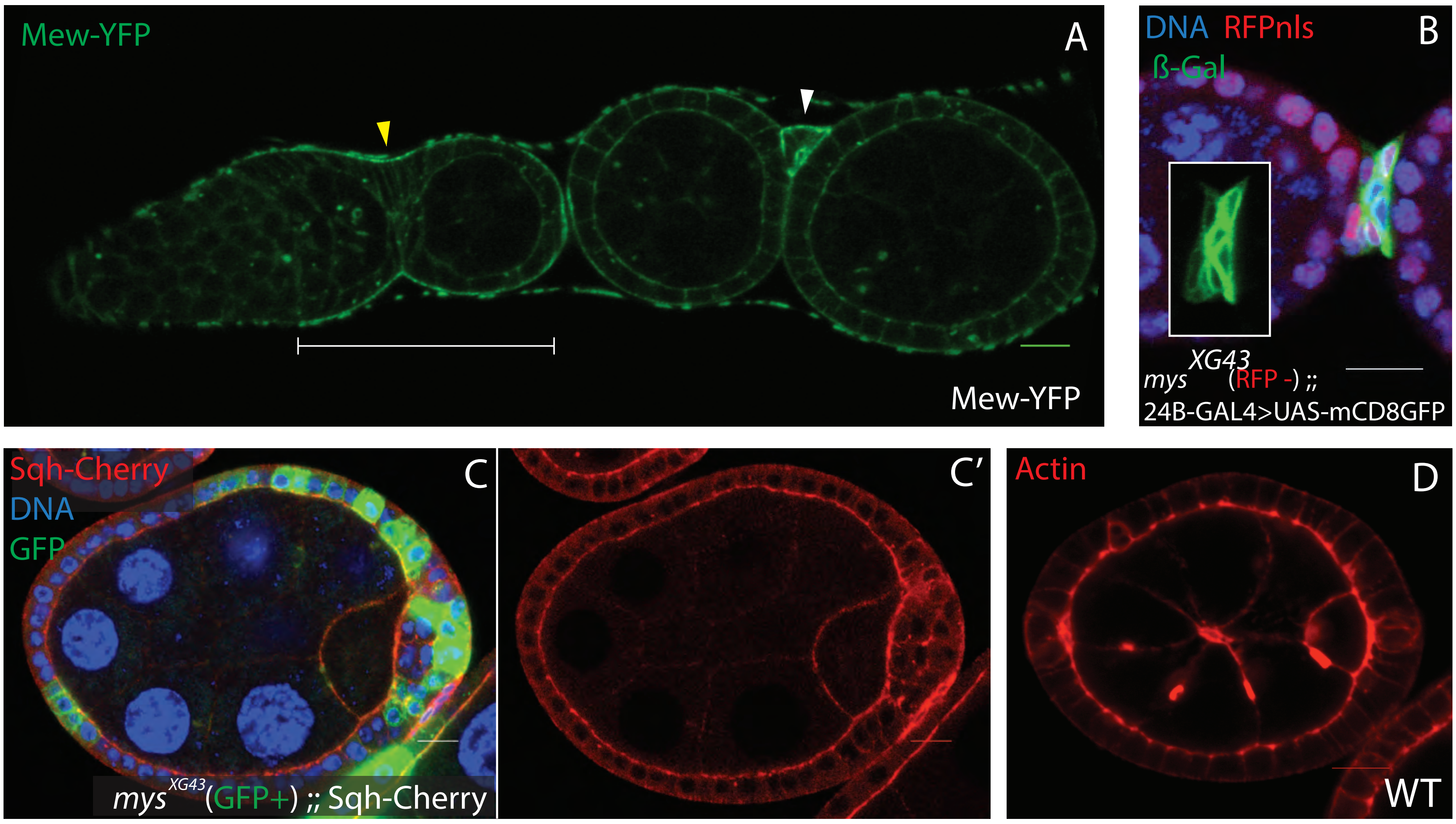

Figure S4

(A) An ovariole expressing Mew-YFP (green) from a BAC genomic construct. Mew is basally enriched in the germarium (bracket) and in the forming (yellow arrow head) and fully produced (white arrow head) interfollicular stalks (n=5).

(B) The stalk region of an ovariole containing *mys*^{XG43} mutant cells marked by the loss of RFP (red) and expressing UAS-LacZ (β-galactosidase, green) and UAS-Flp under the control of the stalk driver 24B-Gal4. Stained for DNA (blue). Although the morphogenesis of the stalk is disrupted, the stalk marker 24B-Gal4 is expressed in *mys*^{XG43} mutant and wild type cells (n=5).

(C) An egg chamber containing *mys*^{XG43} mutant cells marked by the expression of GFP (green) and expressing Sqh-Cherry (red) and stained for DNA (blue). Sqh is expressed and localises (apically enriched) normally in *mys*^{XG43} mutant cells that contact the germline, as well as being expressed at similar levels in cells which do not contact the germline (n=6).

(D) F-actin is apically enriched in the follicle cells of wild-type egg chambers (n=29). All scale bars are 10μM.
